# Nonsteroidal Anti-Inflammatory Drugs and Risk of Melanoma

**DOI:** 10.1155/2011/598571

**Published:** 2011-05-26

**Authors:** Joanne M. Jeter, Joseph D. Bonner, Timothy M. Johnson, Stephen B. Gruber

**Affiliations:** ^1^University of Michigan Cancer Center, 1500 East Medical Center, Ann Arbor, MI 48109, USA; ^2^The Arizona Cancer Center, The University of Arizona, 1515 N Campbell Ave, P.O. Box 245024, Tucson, AZ 85724-5024, USA; ^3^Biomedical Research Informatics Core, Michigan State University, 100 Conrad Hall, East Lansing, MI 48824, USA

## Abstract

Because nonsteroidal anti-inflammatory drugs (NSAIDs) inhibit tumor growth *in vitro*, we investigated the association between NSAIDs and melanoma to determine if there was epidemiologic evidence of a chemopreventive effect from these medications. Three hundred twenty-seven subjects with incident melanoma and 119 melanoma-free controls completed a structured interview assessing melanoma risk factors. The unadjusted odds ratio (OR) for use of nonaspirin NSAIDs was 0.58 (95% CI 0.31–1.11), in a comparison of subjects with melanoma to controls. After adjustment for melanoma risk factors, the OR was 0.71 (95% CI 0.23–2.02). Aspirin users had an unadjusted OR of 0.85 (95% CI 0.45–1.69) and an adjusted OR of 1.45 (95% CI 0.44–4.74). In this pilot study, we found no evidence of a significant association between analgesic use and melanoma risk when potential confounders are assessed. Based on conflicting reports in the literature, meta-analysis may be appropriate.

## 1. Introduction

Melanoma is a potentially fatal tumor that continues to increase in incidence despite public education measures to limit sun exposure [1]. Chemoprevention has been identified as an important strategy for reducing melanoma incidence [2]. Among the candidate agents, nonsteroidal anti-inflammatory drugs (NSAIDs) have been shown to affect the mechanisms of cellular damage caused by ultraviolet radiation [3]. Expression of cyclooxygenase-2 (COX-2), the primary pharmacologic target of NSAIDs, has been reported in up to 93% of melanomas [4]. In addition, *in vitro* and animal studies have demonstrated a protective effect of NSAIDs against melanoma; [4–6] however, studies in humans have had conflicting results [7–9]. 

Using a case-control design, we tested the hypothesis that NSAIDs convey a chemoprotective effect against melanoma.

## 2. Methods

The Genes, Environment, and Melanoma (GEM) study is a multicenter case-control study investigating heredity and environment in melanoma. A full description of the GEM study design has been published separately [10–12]. In this report, only the University of Michigan subjects were used, since supplemental data regarding medication usage was not collected from other centers. 

Individuals with a first primary invasive melanoma diagnosed in the year 2000 and individuals with a second or higher order invasive primary melanoma diagnosed between January 1, 2000, and August 31, 2003, were eligible for participation. At the University of Michigan site, subjects with a first primary melanoma *in situ* diagnosed in 2000 were also enrolled. This site also enrolled spouses of the melanoma subjects to act as controls. The inclusion criteria for controls were a current spousal relationship to a melanoma subject and willingness to provide informed consent to participate. Spouses were excluded from participation as controls if they had a personal history of melanoma. The current analysis is restricted to cases diagnosed with first primary invasive melanoma or melanoma *in situ*.

Participants provided written informed consent at the time of enrollment. The protocol was approved by the Institutional Review Board at the University of Michigan. 

Subjects completed an interview assessing personal and family history of cancer, skin phenotype, medication use, and comorbidities. To assess past and current use of analgesics, they were asked to recall medications used on a daily basis for at least three months. 

Statistical analyses were performed using SAS software (version 9.1). Contingency tables were used to assess the crude associations between medication use and melanoma risk. Fisher's exact test was used to determine *P*-values when cells contained counts of less than 10. Unconditional logistic regression was used to assess the association between medication use and melanoma risk, to adjust for confounding, and to identify any potential effect modification. All reported *P* values are two-sided.

Potential variables evaluated for the multivariate model included age, gender, family history of melanoma, skin color, hair color, eye color, history of severe sunburn, history of cardiovascular disease, history of a musculoskeletal pain condition, tendency of skin to burn, tendency of skin to tan, age at first sunburn, number of moles on the back, smoking history, history of other medical diagnoses (yes/no), and statin use. Covariates were included in the initial multivariate model if the addition of that covariate to the univariate model of analgesic use and melanoma risk resulted in a 10% or greater change in the odds ratio. These covariates were then removed from the saturated multivariate model in a stepwise fashion to optimize the fit to the data as determined by the AIC. 

## 3. Results

Seven hundred twenty-eight individuals were approached for study participation at the University of Michigan. Of these, 509 (69.8%) completed the telephone interview. Melanoma patients accounted for 390 of these subjects; the remaining 119 were melanoma-free spouses of the patients. Among the melanoma subjects, 104 had *in situ* melanoma, 223 had a first (single primary) melanoma, and 63 had a second or higher-order (multiple primary) melanoma. In this analysis, individuals with multiple primary melanomas were excluded, as we could not adjust for selection bias based on survival from the first primary melanoma. Demographic characteristics are described in Table 1, and results are summarized in Table 2. Due to the differences in gender and age between the melanoma and control groups, these variables were empirically included in the multivariate models. Covariates in the final models were age, gender, skin color, family history of melanoma, and number of moles on the back.

### 3.1. Aspirin

Regular use of aspirin was reported by 10.9% of controls. Current or past use of aspirin was associated with an unadjusted odds ratio of 0.85 (95% CI 0.43–1.69). The odds ratio was 1.45 (95% CI 0.44–4.74) in a multivariate model adjusted for age, gender, skin color, family history of melanoma, and number of moles. The number of moles had the most significant effect on the adjusted odds ratio. Restricting the analysis to current users or those with invasive melanoma yielded similar results. Due to small numbers of subjects, past use could not be evaluated. Subjects with *in situ* melanoma were more likely to take aspirin than those with invasive disease, but this difference was not statistically significant (OR 0.615; 95% CI 0.29–1.31).

### 3.2. Nonaspirin NSAIDs

Approximately 14% of control subjects reported regular use of nonaspirin NSAIDs in our study. The unadjusted odds ratio for melanoma risk with any use of nonaspirin NSAIDs was 0.58 (95% CI 0.31–1.11), and the adjusted odds ratio was 0.71 (95% CI 0.23–2.02). Covariates in the multivariate model were the same as in the aspirin model. The addition of aspirin use to the model did not affect the odds ratio, and results were similar when restricted to invasive melanoma. Control subjects were more likely to be current users of NSAIDs (OR 0.45; 95% CI 0.23–0.88); however, this association was no longer significant when adjusted for covariates (OR 0.55; 95% CI 0.19–1.58). Past use was not associated with a decreased melanoma risk. NSAID use in subjects with *in situ* disease was similar to use in those with invasive melanoma.

### 3.3. COX-2 Specific Inhibitors

Approximately 9% of controls reported regular use of COX-2 inhibitors. In an exploratory analysis, the crude odds ratio for melanoma in users of COX-2-inhibitors was 0.61 (95% CI 0.28–1.31), and the adjusted odds ratio was 0.42 (95% CI 0.14–1.27).

### 3.4. Acetaminophen

In order to assess whether the effects of NSAIDs were due to the medical indication rather than the mechanism of action, we evaluated the effect of acetaminophen on melanoma risk. Due to small numbers of users (2.5% of controls), Fisher's exact test was performed and yielded a two-sided *P*-value of  .72.

## 4. Discussion

Our results suggest that aspirin and nonaspirin NSAIDs are not associated with a durable large reduction in risk of melanoma once potential confounders are taken into account. A small beneficial effect of these medications may be seen in a larger study and cannot be excluded. We were unable to fully assess the effects of COX-2 specific inhibitors or acetaminophen due to small numbers of regular users in our study.

The effect of NSAIDS on melanoma has been assessed in four other publications. The first describes a case-control study of 110 women with melanoma and 609 matched controls [7]. The relative risk of melanoma in women taking NSAIDs was 0.45 (95% CI 0.22–0.92). Our study builds on this result with the inclusion of men, an increased number of melanoma cases, and assessment of additional covariates. The second study investigated the effects of NSAIDs in individuals who had previously had melanoma [13]. Individuals taking COX inhibitors were less likely to have new primary melanoma, melanoma recurrence, or melanoma metastasis. Our study takes this question from a secondary/tertiary prevention setting to a primary prevention setting. The third study linked NSAID use in the Vitamins and Lifestyle (VITAL) study to the NCI Surveillance, Epidemiology, and End Results registry [9]. These investigators found no association between NSAID use and melanoma risk. The number of cases in this study is similar to ours; however, many had *in situ* rather than invasive melanomas. Our study expands upon this result by using a larger number of invasive melanoma cases and adding data on number of moles, which was the most significant covariate in our analysis. The fourth study assessed this question in the Dutch PHARMO pharmacy database and the PALGA pathology database [8]. They found a 46% reduction in melanoma risk for women who had continuous use of aspirin (OR 0.54, 95% CI 0.30–0.99). Our work adds melanoma risk factors, which can act as potential confounders. 

Our study has several limitations. Unfortunately, we could not validate reports of medication use with prescription records in our study, as many NSAIDs are dispensed over the counter, and use of these medications is not always accurately collected in the medical record. Sun exposure could not be assessed as a potential confounder due to a lack of a simple, validated measure of this risk factor. However, we did evaluate self-reported history of severe sunburns, age at first sunburn, and tendency to burn as potential confounders; these did not have a significant effect on the odds ratios. Selection bias may be present in the control group, as not all spouses of melanoma subjects participated. Data are not available on nonconsenting spouses, so we are unable to assess whether the participating spouses were representative of the potential control population as a whole. Additionally, the odds ratios may be attenuated by overmatching of spouse controls, who may resemble the cases more than individuals in the general population.

In summary, in our pilot study we found no significant association between analgesic use and melanoma risk when potential confounders are assessed. Based on the conflicting data for this question, evaluation in a larger cohort that also allows for assessment of potential confounders or meta-analysis may be appropriate.

## Figures and Tables

**Table 1:** Demographic characteristics.

Characteristic	Melanoma cases	Controls	*P*-value
All subjects—no	327	119	

Sex—no (%)			
Male	162 (49.5%)	41 (34.5%)	.0047
Female	165 (50.5%)	78 (65.6%)	
Age—yr (median (interquartile range))	53 (41–66)	56 (46–66)	
Family history melanoma—no (%)	51 (15.8%)	11 (9.5%)	.0926
Skin Color—no (%)			
Light	287 (88.9%)	49 (69.0%)	<.0001
Dark	36 (11.1%)	22 (31.0%)	
Number of moles on back (median (interquartile range))	10 (4–29.5)	5 (1–12)	
Current medication use—no (%)	217 (66.4%)	91 (76.5%)	.0413
Past medication use—no (%)	106 (32.9%)	46 (40.0%)	.1918
Comorbidities—no (%)	168 (50.9%)	70 (58.3%)	.1634
Cardiovascular disease—no (%)	37 (8.2%)	9 (7.5%)	.7965
Musculoskeletal pain—no (%)	22 (4.9%)	18 (15.0%)	.0001

SD Standard deviation. *P*-values for categorical variables are from Mantel-Haenszel *χ*2 test.

**Table 2:** Analgesic use and risk of first primary invasive or *In situ* melanoma.

Exposure	Patients	Controls	Total	Crude OR (95% CI)	Adjusted OR (95% CI)
Ever use of aspirin:					
Yes	31	13	44	0.854 (0.431–1.693)	1.447 (0.442–4.736)
No	296	106	402		
Ever use of nonaspirin NSAIDs:					
Yes	29	17	46	0.584 (0.308–1.107)	0.710 (0.234–2.024)
No	298	102	400		
Ever use of acetaminophen:					
Yes	6	3	9	0.723 (0.178–2.937)	Not assessed
No	321	116	437		

OR Odds ratio; CI Confidence interval; NSAIDs Nonsteroidal anti-inflammatory drugs.
